# Early alterations of neurovascular unit in the retina in mouse models of tauopathy

**DOI:** 10.1186/s40478-021-01149-y

**Published:** 2021-03-24

**Authors:** Fan Xia, Yonju Ha, Shuizhen Shi, Yi Li, Shengguo Li, Jonathan Luisi, Rakez Kayed, Massoud Motamedi, Hua Liu, Wenbo Zhang

**Affiliations:** 1 Department of Ophthalmology and Visual Sciences, University of Texas Medical Branch, 301 University Boulevard, Galveston, TX 77555-0144 USA; 2 Department of Pharmacology and Toxicology, University of Texas Medical Branch, Galveston, TX USA; 3 Department of Neurology, University of Texas Medical Branch, Galveston, TX USA; 4 Departments of Neuroscience, Cell Biology and Anatomy, University of Texas Medical Branch, Galveston, TX USA; 5 Institute for Human Infections and Immunity, University of Texas Medical Branch, Galveston, TX 77555 USA

**Keywords:** Retina, Tauopathy, Neurovascular unit, Vascular leakage, Neurodegeneration, Leukocyte adhesion/infiltration, Microglial recruitment/activation, Tau oligomer monoclonal antibody

## Abstract

The retina, as the only visually accessible tissue in the central nervous system, has attracted significant attention for evaluating it as a biomarker for neurodegenerative diseases. Yet, most of studies focus on characterizing the loss of retinal ganglion cells (RGCs) and degeneration of their axons. There is no integrated analysis addressing temporal alterations of different retinal cells in the neurovascular unit (NVU) in particular retinal vessels. Here we assessed NVU changes in two mouse models of tauopathy, P301S and P301L transgenic mice overexpressing the human tau mutated gene, and evaluated the therapeutic effects of a tau oligomer monoclonal antibody (TOMA). We found that retinal edema and breakdown of blood–retina barrier were observed at the very early stage of tauopathy. Leukocyte adhesion/infiltration, and microglial recruitment/activation were constantly increased in the retinal ganglion cell layer of tau transgenic mice at different ages, while Müller cell gliosis was only detected in relatively older tau mice. Concomitantly, the number and function of RGCs progressively decreased during aging although they were not considerably altered in the very early stage of tauopathy. Moreover, intrinsically photosensitive RGCs appeared more sensitive to tauopathy. Remarkably, TOMA treatment in young tau transgenic mice significantly attenuated vascular leakage, inflammation and RGC loss. Our data provide compelling evidence that abnormal tau accumulation can lead to pathology in the retinal NVU, and vascular alterations occur more manifest and earlier than neurodegeneration in the retina. Oligomeric tau-targeted immunotherapy has the potential to treat tau-induced retinopathies. These data suggest that retinal NVU may serve as a potential biomarker for diagnosis and staging of tauopathy as well as a platform to study the molecular mechanisms of neurodegeneration.

**Supplementary Information:**

The online version contains supplementary material available at 10.1186/s40478-021-01149-y.

## Introduction

Tau is a member of the microtubule-associated proteins family, which is mainly expressed by neurons, especially in their axons where it controls the polymerization and stabilization of the microtubules and regulates axonal transport. Tauopathies, characterized by abnormal intracellular accumulation of aggregated and/or hyperphosphorylated tau within neurons, is a hallmark of Alzheimer's disease (AD) and a number of other disorders including frontotemporal dementia with parkinsonism-17 (FTDP-17), Pick disease, progressive supranuclear palsy and corticobasal degeneration [5, 21]. Tauopathies are among the most crippling conditions that affect our rapidly growing aging population. Due to the lack of effective diagnostics and treatments, these diseases significantly impair the daily life of patients and markedly impose financial costs to them, their family members and society.

The retina comprises several cell types including retinal neurons, vascular cells, microglia and glial cells. It is an extension of the neural network of the brain and shares many similar pathophysiological changes and underlying mechanisms with the brain during neurodegenerative diseases including AD [8, 12, 17, 18, 20, 25, 33–35, 47, 57, 64]. Since the retina is not wrapped by the skull, it is much easier to observe the retina than the brain noninvasively. Additionally, due to the anatomical and functional characters, the pathological changes in the retina may precede those in the brain. These features make the retina an appealing source of noninvasive biomarkers as well as an alternative platform to study neurovascular coupling in tauopathy [64]. Although previous studies have some exploration of retinal abnormality in different AD-related models [8, 12, 18, 20, 25, 33, 47, 58, 61], retinal vascular changes in this process are largely unknown; and quantitative and temporal analyses for the alterations of different retinal cell types are missing.

P301S and P301L tau gene mutations are two of the most frequently studied tau mutations linked to FTDP-17 [24, 65]. P301S and P301L transgenic mice closely recapitulate many features of human tau-associated pathology including the formation of filamentous tau lesions, synapse loss and neurodegeneration [31, 38, 65]. Therefore, these mouse strains have been widely used as models to study mechanisms and treatments of tauopathy including AD [4, 10, 37, 60, 65]. Here we utilized P301S and P301L mice to perform comprehensive and longitudinal analyses of changes in the retinal neurovascular unit (NVU) during tauopathy. We found that retinas underwent vascular inflammation and barrier breakdown, retinal ganglion cell (RGC) dysfunction and degeneration, and Müller cell gliosis, associated with the progression of tau pathology. Moreover, vascular inflammation and barrier breakdown even occurred in 1-month-old P301S mice, which preceded manifest neuron degeneration. More importantly, retinal pathology in P301L mice was attenuated by the treatment with a tau oligomer monoclonal antibody (TOMA) which has been used to treat tauopathy in the brain [10], further supporting the notion that the retina and brain share common pathological mechanisms during tauopathy.

## Materials and methods

### Animals

C57BL/6J wild type (WT) mice (Stock No: 000664) were originally obtained from the Jackson Laboratory (Bar Harbor, ME) and subsequently bred in the animal care facility at the University of Texas Medical Branch. P301S hemizygous mice on a C57BL/6J and C3H/HeJ mixed background (Stock No: 008169) were purchased from the Jackson Laboratory and bred with C57BL/6J mice to generate P301S and WT littermates. Cx3cr1^GFP/+^mice (Stock No: 005582) were from the Jackson Laboratory and maintained by crossing with C57BL/6J mice. To generate P301S; Cx3cr1^GFP/+^ mice, P301S mice were crossed with Cx3cr1^GFP/+^ mice. P301L homozygous mice on a C57BL/6, DBA/2, SWR/J mixed background (Model 2508) were purchased from Taconic Biosciences (Rensselaer, NY) and crossed with C57BL/6J mice for two generations. Then hemizygous P301L mice were intercrossed to generate homozygous P301L and background-matched WT mice. The homozygous and its corresponding WT colonies were maintained by incrossing homozygotes and WT mice, respectively. Since many transgenic mice used to study tauopathy or AD pathology are on a mixed background potentially bearing inherited retinal degeneration mutations [11], tail snips from founder mouse lines (P301S, Cx3cr1^GFP/+^ and P301L) were sent to GenoTyping Center of America (Waterville, ME) to perform single nucleotide polymorphism (SNP) before using them to set up colonies to produce mice used for the experiments. SNP analysis demonstrated these mice did not bear Pde6b^rd1^ mutation which is often associated with C3H and SWR background and Crb1^rd8^ mutation which is often associated with C57BL/6N background.

To study the effects of TOMA treatment, mouse TOMA (MABN819, MilliporeSigma, Burlington, MA) or IgG isotype control (31903, ThermoFisher Scientific, Waltham, MA) were injected to P301L mice (i.v., 30 μg/mouse) [10] at 1 month of age and 1 week before sample collection at 3 months of age. Mice were maintained on a 12:12 light/dark cycle with food and water available ad libitum. All experiments procedures and use of animals were approved by the Institutional Animal Care and Use Committee of the University of Texas Medical Branch.

### High resolution spectral domain optical coherence tomography (SD-OCT)

P301S, P301L mice and age- and strain-matched WT mice were examined by OCT before sacrifice. Briefly, mice were anesthetized via intraperitoneal injection with a combination of ketamine (100 mg/kg) and xylazine (10 mg/kg). Mouse eyes were topically dilated with one drop of tropicamide and phenylephrine. Eyes were imaged using Spectral Domain Ophthalmic Imaging System (Envisu R2200, Bioptigen Inc., NC) as described previously [41–43]. Annular scans consisted of 1000 A-scans × 100 B-scans covering a donut-shaped area centered at the optic nerve disc were performed in order to remove variance of optic nerve head measurement. The inner and outer radiuses of the donut-shaped area were 200 and 700 μm from the center of the optic nerve disc, respectively. Then OCT depth thickness report and analysis were generated from Bioptigen’s automated segmentation algorithm developed for the murine eye. The thickness of each retinal layer presented in the report for each eye was an average of 100,000 measurements (A-scans) in the donut-shaped area, which accurately measured retinal thickness and excluded the necessity to register and quantify the same anatomical landmark between different animals. The ganglion cell complex (GCC) includes all three innermost layers: the nerve fiber layer (NFL), the ganglion cell layer (GCL) and the inner plexiform layer (IPL).

### In vivo imaging by scanning laser ophthalmoscopy (SLO)

Mice were anesthetized via intraperitoneal injection with a combination of ketamine (100 mg/kg) and xylazine (10 mg/kg). After eyes were topically dilated with one drop of tropicamide and phenylephrine, mice were properly located on the platform. The Heidelberg Spectralis HRA system (Heidelberg Engineering, Franklin, MA) was used to image GFP^+^ cells within the context of the retinal tissue at 488-nm excitation wavelength for GFP fluorescence [26].

### Dark-adapted electroretinography (ERG) analysis

ERG analysis was performed as described previously [26, 41, 43]. Briefly, mice were dark-adapted overnight  and anesthetized via intraperitoneal injection with a mixture of ketamine (100 mg/kg) and xylazine (10 mg/kg). Mouse pupils were topically dilated with a mixture of atropine and phenylephrine and the corneas were kept moist with the application of Celluvisc. Next, mice were placed on a self-heating platform to maintain a constant body temperature of 37.0 °C, gold ring electrodes were placed on the surface of the cornea, and ERG responses were measured using the Espion system (Diagnosys LLC, Lowell, MA). pSTRs were recorded in response to a series of white flashes with intensities ranging from − 4.3 to − 3.2 log cd s/m^2^. Each record was an average of at least 50 responses.

### Leukostasis

Retinal leukostasis assay, labeling adherent leukocytes to retinal vasculature, was performed as described previously [41, 43]. Briefly, at 1, 3, 8 months of age, mice were anesthetized via intraperitoneal injection with a mixture of ketamine (100 mg/kg) and xylazine (10 mg/kg). After the chest cavity was opened and a 20-gauge perfusion cannula was introduced to the left ventricle, the right atrium was cut open for outflow. Phosphate-buffered saline (PBS) was perfused through left ventricle to remove erythrocytes and nonadherent leukocytes, which was followed by perfusion with rhodamine-coupled concanavalin A (ConA) lectin (40 μg/mL in PBS, pH 7.4; Vector Laboratories, Burlingame, CA) to label adherent leukocytes and vasculature. Subsequently, PBS was perfused again to remove residual unbound Con A. After eyeballs were collected and fixed with 4% paraformaldehyde (PFA) overnight, retinas were dissected and stained with anti-CD45 antibody (1:400, 550539, BD Biosciences, San Jose, CA). Leukocytes inside the blood vessels (leukostasis) are ConA^+^CD45^+^ (red and green fluorescence), while leukocytes outside the blood vessels (leukocytes infiltrated into the retina) are ConA^−^CD45^+^ (only green fluorescence). The total number of adherent leukocytes per retina and leukocytes infiltrated into the retina per retina were counted [41, 43]. Of note, although suboptimal concentration of CD45 antibody was used for staining (1:400 dilution rather than 1:10–1:50 dilution recommended by the company) and only cells with round shape were counted, there was possibility that very few microglia that had relative high CD45 expression and were round might be counted as infiltrated leukocytes.

### Western blot

Whole retinas were homogenized and lysed for 30 min on ice in lysis buffer (50 mM Tris–HCl, pH 7.4, 150 mM NaCl, 0.25% deoxycholic acid, 1% NP-40, and 1 mM EDTA) supplemented with Complete Protease and Phosphatase Inhibitors (Roche Applied Science, Indianapolis, IN). After retinal lysates were centrifuged (14,000 rpm, 15 min, 4 °C), protein concentration was assessed with Pierce BCA Protein Assay Kit (Pierce, Rockford, IL). 10 μg protein per sample was electrophoresed in a 10% SDS-PAGE gel, and electroblotted onto nitrocellulose membranes. After blocking, the membranes were incubated with primary antibody against tau (Tau-5, ab80579, Abcam, Cambridge, MA) overnight at 4 °C. After washes, the membranes were incubated for 1 h at room temperature with HRP-conjugated secondary antibody (1:2000; Cytiva, Marlborough, MA). After washing, proteins were detected by enhanced chemiluminescence (Pierce) using Bio-Rad ChemiDoc XRS + (Bio-Rad Laboratories, Hercules, CA).

### Immunostaining

For retinal flatmounts, eyeballs were fixed in 4% PFA at 4 °C overnight. Retinas were then dissected, washed with PBS, and blocked and permeabilized with PBS containing 5% normal goat serum and 0.3% Triton-X-100 for 3 h. For occludin and VE-Cadherin, eyeballs were fixed in 4% PFA for 30 min at room temperature, and retinas were treated with methanol for 20 min at − 20 °C. After blocking, retinas were incubated with antibodies against Iba1 (1:200, 019-19741, FUJIFILM Wako Chemicals, Richmond, VA), Tuj1 (1:400, 801202, BioLegend, San Diego, CA), Opsin 4 (1:200, PA1-780, Invitrogen, Carlsbad, CA), occludin (1:200, 71-1500, ThermoFisher Scientific) or VE-Cadherin (1:200, 550548, BD Biosciences) at 4 °C overnight. After washing, retinas were incubated with Alexa Fluor 488 or 594-conjugated secondary antibodies (1:400, Invitrogen) at 4 °C for 4 h. Finally, retinas were mounted, and images were taken by confocal microscopy (LSM 800, Carl Zeiss Inc, Thornwood, NY). For cell counting, eight non-overlapping images were taken at the peripheral or middle region of each retinal flatmounts at 200 × magnification, then cells were manually counted and averaged for each sample. Microglial soma size and roundness and nearest neighbor distance (NND) were quantified using methods and formulas developed by Davis et al. [15].

For retinal cryosections, immunostaining was performed as described previously [41, 43]. The following antibodies were used: anti-phospho-tau (Thr231) monoclonal antibody (AT180) (1:500, MN1040, Invitrogen), anti-phospho-tau (Ser202, Thr205) monoclonal antibody (AT8) (1:500, MN1020, Invitrogen), anti-tau polyclonal antibody (1:800, A002401-2, Agilent Technologies, Santa Clara, CA), anti-GFAP (1:500, Z033401-2, Agilent Technologies). Cells were counterstained with DAPI. Fluorescent images were taken by confocal microscopy.

### Permeability assay to measure vascular leakage

Leakage of albumin into the retina was measured as reported previously [39] with some modifications. Briefly, after mice were anesthetized via isoflurane, mice were injected with FITC-bovine serum albumin (BSA) (*i.v.*, 100 mg/kg body weight, Sigma-Aldrich, St. Louis, MO). After circulation for 1 h (whole retina) and 20 min (retinal sections), mice were euthanized. To assess retinal vascular leakage in whole retina, whole blood was obtained from the right ventricle and centrifuged at 2000*g* for 15 min for plasma, which was diluted 100 times with 1 × PBS. Next, mice were perfused via the left ventricle with PBS to remove intravascular blood. The whole retinas were isolated from eyeballs carefully to avoid contamination of aqueous humor, homogenized with RIPA lysis buffer (20-188, MilliporeSigma, Burlington, MA), and centrifuged at 16,600*g* for 15 min. Subsequently, fluorescence intensity in the supernatant from the retinal homogenate and diluted plasma were measured by Synergy H1 Hybrid Multi-Mode Reader (BioTek, Winooski, VT) with excitation at 485 nm and emission at 528 nm. Retinal homogenate and diluted plasma from mice without FITC-BSA injection were used as blank. Finally, fluorescence intensity in the retina was adjusted by retinal weight and the fluorescence of the plasma and normalized to retinas from WT mice. To assess vascular leakage in retinal sections, eyeballs were fixed in ice-cold 4% PFA for 1 h and then dehydrated in 30% sucrose 4° C overnight, finally, embedded in optimal cutting temperature compound. Retinal cryosections were cut and images were taken by fluorescence microscopy [1]. Leakage of albumin was quantified by measuring the fluoresce intensity of FITC-BSA from the inner plexiform layer (IPL), inner nuclear layer (INL), and outer plexiform layer (OPL) of the neural retina using ImageJ (National Institutes of Health, Bethesda, MD).

### Statistics

Data were presented as mean ± standard error of mean (SEM) and analyzed by Student’s t-test. Statistical analysis was conducted using GraphPad Prism program (GraphPad Software Inc., La Jolla, CA). A *p* value < 0.05 was considered statistically significant.

## Results

### Characterization of tau accumulation in the retinas of P301S mice

**Fig. 1:**
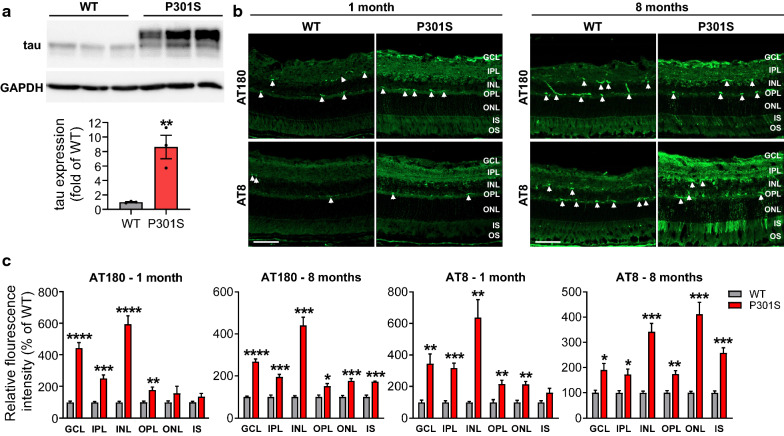
Total and phosphorylated tau are increased in the retina of P301S mice. **a** Retinal lysates from 3-month-old WT and P301S mice were blotted with anti-tau (Tau-5) for total tau. GAPDH was used as internal control. Graph represents densitometric analysis of tau protein normalized to GAPDH. n = 3/group. **b** Retinal sections from 1 and 8-month-old WT and P301S mice were stained with AT180 and AT8 antibodies for phosphorylated tau (green). Arrowheads indicate non-specific staining. **c** Quantification of fluorescence intensity of AT180 and AT8 in individual retinal layers. Non-specific staining was removed when performing quantification. Scale bar: 50 µm. n = 4/group. *p < 0.05; **p < 0.01; ***p < 0.001; ****p < 0.0001 versus WT. *GCL* ganglion cell layer, *IPL* inner plexiform layer, *INL* inner nuclear layer, *OPL* outer plexiform layer, *ONL* outer nuclear layer, *IS* inner segment, *OS* outer segment

Tau P301S transgenic mice overexpress human tau with a P301S mutation in exon 10 [56, 65]. In the retina of P301S mice from the PS19 line, human tau protein overexpression was examined by western blot using Tau-5 antibody (Fig. 1a). There was a remarkable increase of total tau expression in transgenic mice compared with WT mice. To investigate the location of phosphorylated tau in the retinas of WT and P301S mice, we performed immunostaining with antibodies against tau phosphorylation at Thr231 (AT180) and Ser202/Thr205 (AT8), which are critical for tau's hyperphosphorylation and aggregation [30, 40, 49]. We observed that phosphorylated tau, localized in different retinal layers, was significantly increased in the retina of P301S mice. While the pattern of AT180 staining was slightly changed between 1- and 8-month-old P301S retinas, AT8 staining was robustly increased in the outer nuclear layer (ONL) and photoreceptor inner segment (IS) of 8-month-old P301S retinas, suggesting the progression of tauopathy during aging (Fig. 1b, c).

### Retinal edema and vascular leakage in the retinas of P301S mice

**Fig. 2:**
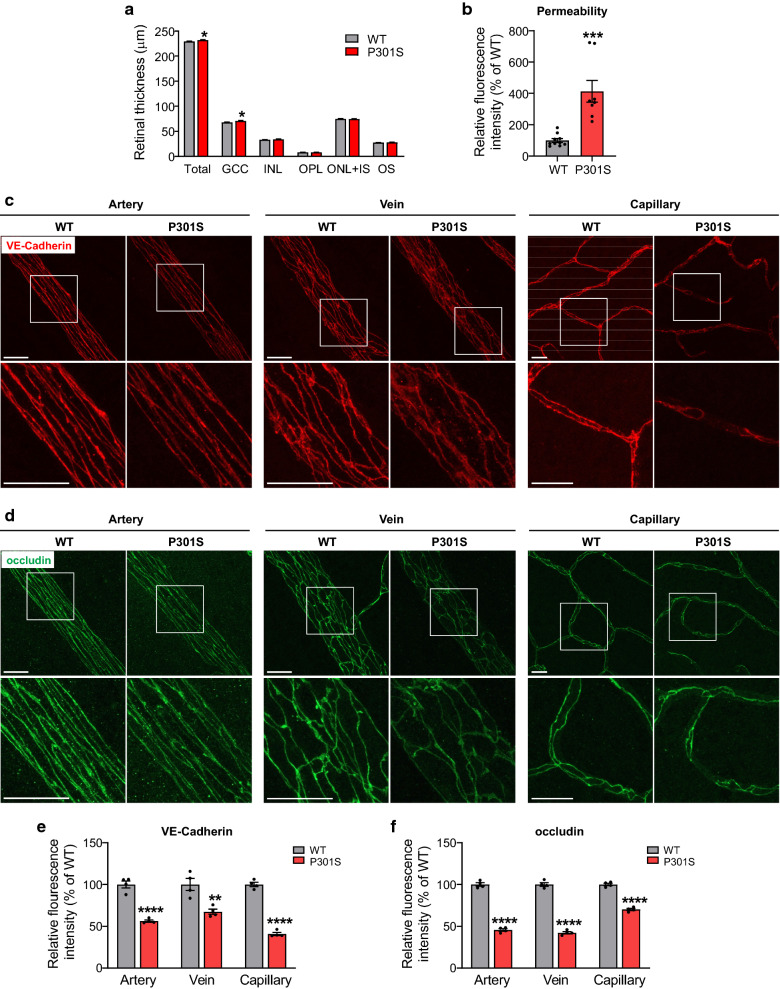
Blood–retina barrier integrity is impaired in the retina of P301S mice. **a** Bar graph represents the OCT analysis of thickness of total retina and individual retinal layers. n = 22/group. **b** Permeability assay. FITC-BSA was intravenously injected into 1-month-old WT and P301S mice. 1 h after injection, blood from circulation was removed by PBS perfusion, and albumin leakage from vessels to neuronal retina was measured by quantifying the fluorescence intensity in retinal homogenates and normalized to that of in the plasma. The normalized fluorescence intensity in WT mice was used as the reference. n = 8–10/group. **c**, **d** Representative images of the adherens junction protein VE-Cadherin and tight junction protein occludin in retinal flatmounts from WT and P301S mice at 1 month of age. Squares in the upper panel of images are zoomed in to show vascular integrity. Scale bar: 20 μm. n = 4/group. **e**, **f** Quantification of fluorescence intensity of VE-Cadherin and occludin. *p < 0.05; **p < 0.01; ***p < 0.001; ****p < 0.0001 versus WT. *GCC* ganglion cell complex, including all three innermost layers: nerve fiber layer, ganglion cell layer and inner plexiform layer, *INL* inner nuclear layer, *OPL* outer plexiform layer, *ONL* outer nuclear layer, *IS* inner segment, *OS* outer segment

To characterize retinal abnormality during the early stage of tauopathy, we analyzed the changes of retinal structure of P301S mice compared with WT littermate controls at 1 month of age. We used optical coherence tomography (OCT) to non-invasively measure the thickness of each retinal layer in live mice, and found the thickness of total retina and ganglion cell complex (GCC) of P301S mice was thicker than that of age-matched WT mice (Additional file 1: Fig. S1 and Fig. 2a). The increase of retinal thickness observed in OCT was likely caused by retinal edema due to increased vascular leakage. To test this possibility, we performed permeability assay by injecting FITC-BSA and measured albumin leakage from vessels to the neural retina (Fig. 2b). We found that FITC-BSA fluorescence intensity was significantly increased in the retinas of P301S mice. Since vascular integrity and permeability barrier function is greatly dependent on the dynamics of cell–cell adherens and tight junctions between endothelial cells, we performed immunostaining for VE-cadherin, a component of endothelial cell–cell adherens junctions, and tight junction protein occludin, in retinal flatmounts of both P301S and WT littermates. We observed that WT mice exhibited strong, sharp and continuous staining of VE-Cadherin and occludin in retinal vessels, whereas P301S mice exhibited weaker, more diffuse and discontinuous staining at cell margins in retinal vessels (Fig. 2c–f). These results indicate that the integrity of retinal vasculature is disrupted in tauopathy, leading to breakdown of blood–retinal barrier which occurs as early as 1 month of age.

### Increased vascular inflammation in the retinas of P301S mice

**Fig. 3:**
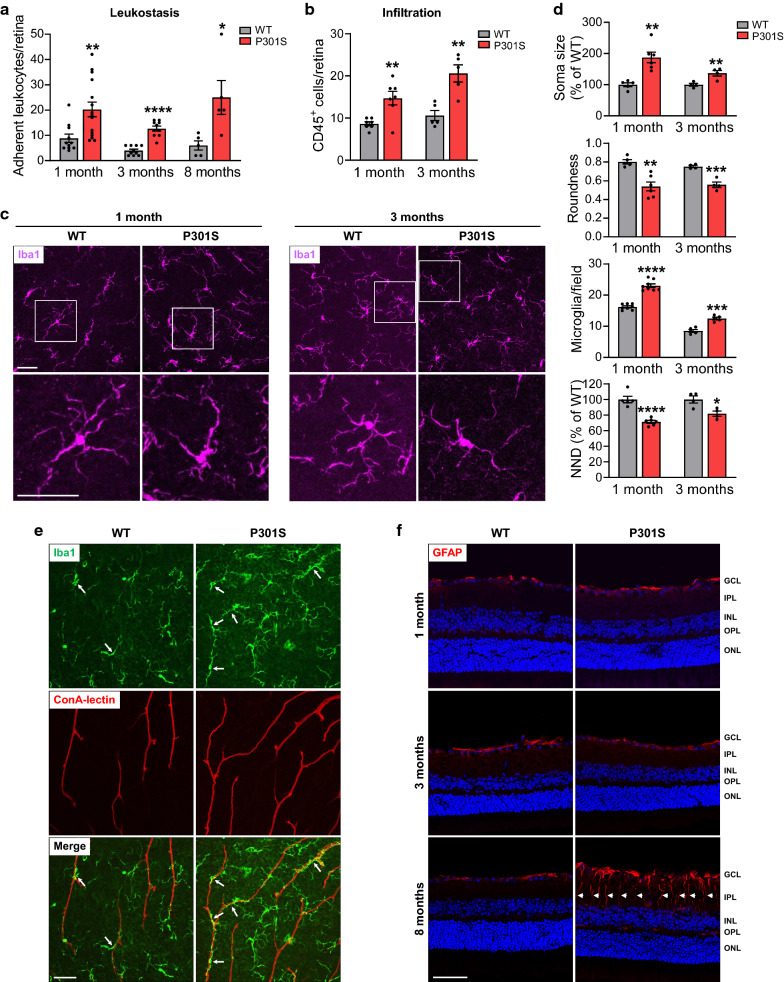
Leukocyte adhesion/infiltration, microglial recruitment/activation and gliosis are increased in the retina of P301S mice. **a** WT and P301S mice were subjected to leukostasis assay at various ages. Bar graph represents the number of leukocytes adherent to the retinal vasculature per retina. n = 5–15/group. **b** Leukocytes were stained with anti-CD45 antibody in retinal flatmounts of WT and P301S mice, and infiltrated leukocytes in the retina were quantified. n = 5–7/group. **c** Microglia were stained with anti-Iba1 antibody (purple) at 1 and 3 months of age. Images were taken at the NFL-GCL by confocal microscopy. Squares in the upper panel of images are zoomed in to more clearly show microglial activation. **d** Bar graphs represent the quantification of morphological parameters of microglia at the NFL-GCL, including soma size and roundness, number and nearest neighbor distance (NND). n = 4–8/group. **e** Microglia were stained with anti-Iba1 antibody (green) and vasculature was co-labeled with ConA-lectin at 1 month of age. Images were taken at the NFL-GCL by confocal microscopy to show the relationship of microglia and vasculature. Arrows indicate those microglia close to vessels. n = 3/group. **f** The activation of Müller cells was assessed by immunostaining with antibody against GFAP (red) in retinal sections. Arrowheads indicate activated Müller cells. Blue staining indicates nuclei. n = 3–5/group. Scale bar: 50 µm. *p < 0.05; **p < 0.01; ***p < 0.001; ****p < 0.0001 versus WT. *GCL* ganglion cell layer, *IPL* inner plexiform layer, *INL* inner nuclear layer, *OPL* outer plexiform layer, *ONL* outer nuclear layer

Vascular inflammation plays a key role in vascular leakage and tissue swelling. We then quantified leukocytes adhesion to the wall of retinal vessels, which is the first step of inflammation, by leukostasis assay in WT and P301S mice at different ages. In this assay, we used concanavalin A (Con A) to perfusion-label vessels and leukocytes attached to vessels (Additional file 1: Fig. S2). Quantification of adherent leukocytes in retinal flatmounts showed significant differences between WT and P301S mice at all analyzed time points (Fig. 3a), suggesting that vascular inflammation is developed in the retina of P301S mice. With the progression of the disease, the extent of vascular inflammation became severer as indicated by increased fold-change of adherent leukocytes at 3 and 8 months of age (2.3 fold at 1 month of age, 3.2 fold at 3 months of age, and 4.2 fold at 8 months of age) (Fig. 3a). In the meanwhile, we stained the retina with CD45 antibody to identify infiltrated leukocytes and found that its number was also significantly increased in P301S mice at 1 and 3 months of age (Fig. 3b).

### Activation of microglia in the retinas of P301S mice

In addition to leukocytes, microglia play an important role in retinal inflammation by functioning as resident innate immune cells. In the brain, microglia-mediated neuroinflammation is a key player in neuronal injury in neurodegenerative diseases including tauopathies. Therefore, we subsequently investigated changes of microglia in P301S and age-matched WT mice at different stages of tauopathy. Retinal flatmounts from WT and P301S mice were labeled with anti-Iba1 antibody and analyzed for alterations of average number and morphology (Fig. 3c, d; Additional file 1: Fig. S3). In both WT and P301S mice, the cell bodies of microglia were distributed in three different layers in the inner retina: nerve fiber layer (NFL)-GCL, IPL and OPL. In the NFL-GCL of WT retina, microglia existed in a resting state with a highly ramified morphology. In contrast, as early as 1 month of age, the microglia in the NFL-GCL of P301S retina underwent morphological shift, transforming from a resting state into an activated state [15], characterized by process retraction and thickening, soma enlargement with an amoeboid shape (reduced roundness) (Fig. 3c, d). Moreover, the average number of microglia located in the NFL-GCL was significantly increased, accompanied by decreased nearest neighbor distance (NND) in P301S retinas compared with their age-matched WT retinas at both 1 and 3 months of age (Fig. 3c, d). Of interest, in P301S retina, more microglia tended to adhere to vessels without obvious change in the morphology of vessels (Fig. 3e). In contrast to the microglia in the NFL-GCL, most of microglia in the IPL and OPL were ramified, and the number and morphology of them were indistinguishable between P301S mice and age-matched WT mice (*p* = 0.1660 and 0.6366 for 1 month of age; *p* = 0.6843 and 0.6549 for 3 months of age) (Additional file 1: Fig. S3). Overall, these data indicate that microglial recruitment and activation occur in the retina of P301S mice as early as at 1 month of age.

The significant change of microglia in the NFL-GCL of P301S mice prompted us to test the feasibility of examining early retinal change during tauopathy by non-invasive imaging. Cx3cr1^GFP^ transgenic mice were generated by inserting an enhanced green fluorescent protein (GFP) gene into exon 2 of the *Cx3cr1* gene, therefore GFP is highly expressed on microglia [29]. We crossed this strain with P301S mice to label microglia with green fluorescence and used confocal scanning laser ophthalmoscope (SLO) to examine microglia in the retina of live mice. Consistently, SLO imaging revealed a significant increase in microglial number in the P301S retina (Additional file 1: Fig. S4). This result suggests that non-invasive imaging of microglia could be potentially used to diagnose early tauopathy.

### Müller cell gliosis in the retinas of P301S mice

Müller cells span all retinal layers, constitute the principal glial cells of the retina and provide support to retinal neurons. Müller cell gliosis, indicated by increased expression of glial fibrillary acidic protein (GFAP), is an indicator of retinal injury [7]. To examine Müller cell gliosis in relation to tauopathy, we assessed GFAP expression in retinal sections at 1, 3 and 8 months of age. At 1 and 3 months of age, GFAP expression was restricted to astrocytes and Müller cell end feet in the NFL in both WT and P301S mice, and there was no significant difference in the expression of GFAP between these two strains of mice (*p* = 0.4496 and 0.0954) (Fig. 3f; Additional file 1: Fig. S5A). However, at 8 months of age, the expression of GFAP in P301S mice was no longer limited to astrocytes and the end feet of Müller cell in the NFL but extended throughout the whole length of Müller cells (Fig. 3f) and the number of activated Müller cells was significantly increased (Additional file 1: Fig. S5A). These results suggest that Müller cell gliosis occurs at a relative later time point during tauopathy. Interestingly, phosphorylated tau was observed in activated Müller cells (Additional file 1: Fig. S5B, C), suggesting phosphorylated tau was uptaken by glial cells.

### RGC degeneration and dysfunction in the retinas of P301S mice

**Fig. 4:**
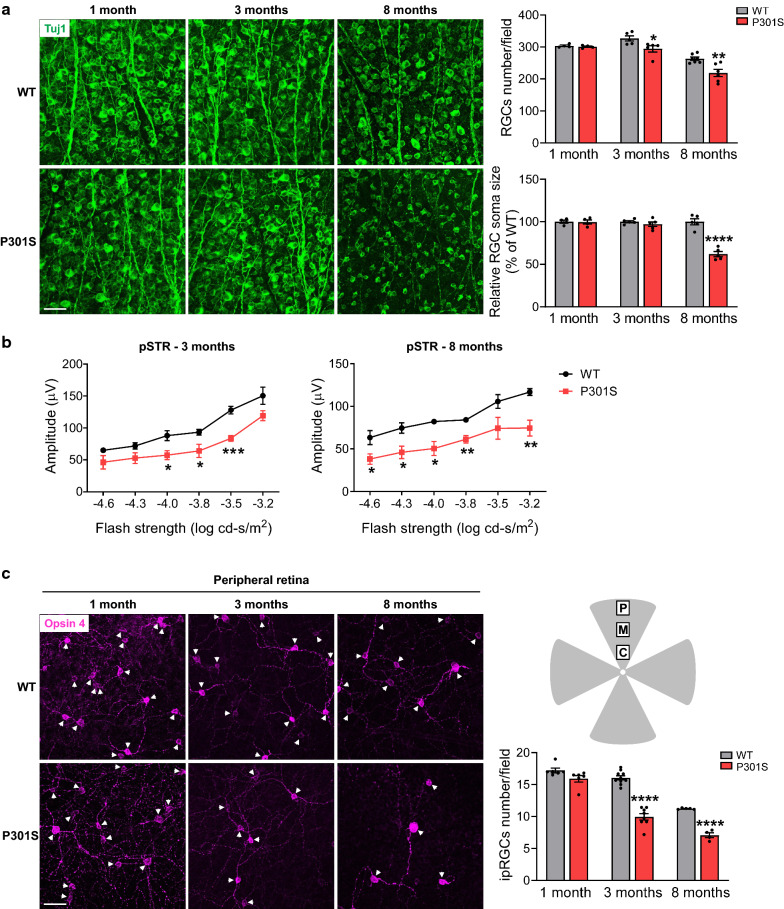
RGCs and ipRGCs are impaired in the retina of P301S. **a** RGCs were stained with anti-Tuj1 antibody (green) in retinal flatmounts from WT and P301S mice at 1, 3 and 8 months of age. Bar graphs represent the number and soma size of RGCs per field. n = 4–7/group, eight images were taken at the peripheral retina for each sample and calculated as average value. **b** ERG analysis (pSTR) over a range of stimulus strengths in WT and P301S mice at 3 and 8 months of age. n = 4–5/group. **c** ipRGCs were stained with Opsin 4 antibody (purple) and the number of ipRGC at the peripheral retina was quantified. Arrowheads indicate the soma of ipRGCs. Schema depicts retinal area selected to take images. *P* peripheral area, *M* middle area; *C* central area. n = 4–10/group; eight images were taken at the peripheral retina for each sample and calculated as average value. Scale bar: 50 µm. *p < 0.05; **p < 0.01; ***p < 0.001; ****p < 0.0001 versus WT

RGCs, the only output neurons of the retina, receive visual information from photoreceptors via bipolar cells and amacrine cells and send it to the brain through their long axons. To quantitatively determine the loss of RGCs in mouse models of tauopathy, retinas from P301S mice at 1, 3 and 8 months of age were collected and stained with the RGC-specific marker anti-Tuj1 to assess RGC density (Fig. 4a). P301S mice displayed no significant loss of RGCs compared with WT mice at 1 month of age (*p* = 0.4031). However, with the development of tauopathy, P301S mice underwent detectable and more dramatic decreases in total RGC number at 3 and 8 months of age. In addition, in the retinas of 8-month-old P301S mice, the soma size of RGCs was reduced dramatically, indicating that the remaining RGCs in P301S mice might be in a vulnerable situation.

To assess the function of RGCs during the development of tauopathy, we used dark-adapted (scotopic) ERG to record positive scotopic threshold response (pSTR) that reflects the functions of RGCs [26, 41]. This analysis showed that the amplitudes of pSTR were significantly reduced in P301S mice at 3 and 8 months of age compared with WT mice (Fig. 4b), which is consistent with the aforementioned alterations of RGCs in terms of cell number and morphology, suggesting RGCs are undergoing dysfunction/degeneration during tauopathy**.**

### Loss of ipRGCs in the retinas of P301S mice

Intrinsically photosensitive RGCs (ipRGCs), as non-rod and non-cone photoreceptors, account for approximately 1% of RGCs in mouse retina [16]. ipRGCs can sense the light due to self-carried melanopsin and play a main role in synchronizing circadian rhythms. Since the progression of AD is often associated with circadian dysfunction [48], we wondered if ipRGCs behaved differently compared to total RGC population. To examine ipRGCs in tauopathy, retinas from WT and P301S mice at 1, 3 and 8 months of age were collected and stained with an antibody against ipRGC-specific marker melanopsin (Fig. 4c; Additional file 1: Fig. S6). We observed that the density of ipRGCs was higher in the peripheral area than in the middle area of both WT and P301S retinas. At 1 month of age, although the density of ipRGCs in the peripheral retina of P301S mice was indiscernible from that of WT littermates (Fig. 4c , *p* = 0.0639), their density was significantly decreased in the middle area of the retina of P301S mice (Additional file 1: Fig. S6). At 3 and 8 months of age, both the middle and peripheral area of P301S retinas showed significant loss of ipRGCs (Fig. 4c; Additional file 1: Fig. S6). Most importantly, the loss of ipRGCs was more robust than the loss of Tuj1-stained RGCs at both 3 and 8 months of age, suggesting ipRGCs are more vulnerable to tau accumulation than other RGCs in the retina.

### Retinal pathology in P301L mice

**Fig. 5:**
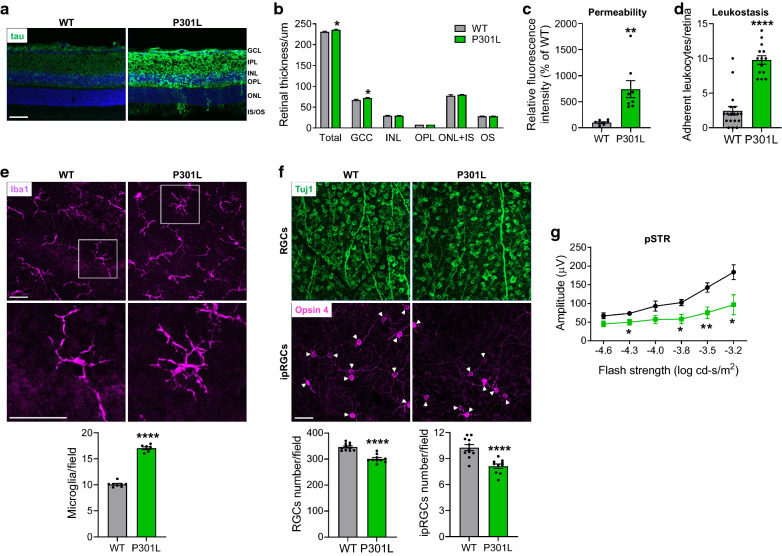
Characterization of retinal pathogenesis in P301L mice at 3 months of age. **a** Total tau (green) was stained with tau antibody in retinal sections from 3-month-old WT and P301L mice. n = 4/group. **b** OCT analysis of retinal thickness. n = 20–26/group. **c** Retinal permeability. n = 8/group. **d** Leukostasis. Bar graph represents the number of stationary leukocytes adherent to the retinal vasculature per retina. n = 14–18/group. **e** Microglia were stained with anti-Iba1 antibody (purple) and images were taken at the NFL-GCL by confocal microscopy. Squares in the upper panel of images are zoomed in to show microglial activation. Bar graph represents the number of microglia at the NFL-GCL. n = 7–8/group. **f** Representative images of retinal flatmounts labeled with Tuj1 antibody (green) for RGCs and Opsin 4 antibody (purple) for ipRGCs at the peripheral retina. Arrowheads indicate the soma of ipRGCs. Bar graphs represent the numbers of RGCs and ipRGCs per field. n = 10–11/group; eight images were taken at the peripheral retina for each sample and calculated as average value. **g** ERG analysis (pSTR) over a range of stimulus strengths. n = 6/group. Scale bar: 50 µm. *p < 0.05; **p < 0.01; ****p < 0.0001 versus WT

To further confirm our findings in P301S mice, we analyzed retinal pathologies in P301L mice that carry a transgene of human tau with the P301L mutation, which is another mutation causing tauopathy [31, 38]. With immunostaining, we demonstrated that human P301L tau was overexpressed in different retinal layers (Fig. 5a). Similar to P301S mice, the thickness of total retina and GCC, which was examined by OCT, was increased in P301L mice (Fig. 5b). Retinal vascular leakage determined by FITC-BSA permeability assay (Fig. 5c), inflammation assessed by leukostasis (Fig. 5d) and microglial activation/recruitment in the NFL-GCL but not in the IPL and OPL (*p* = 0.8678 and *p* = 0.9378) (Fig. 5e; Additional file 1: Fig. S7A) were significantly increased in 3-month-old P301L mice, while the numbers of RGCs and ipRGCs were significantly decreased (Fig. 5f; Additional file 1: Fig. S7B), associated with impaired RGC function (Fig. 5g). These data indicate that retinal vascular and neuronal pathological changes also occur in P301L mice at the early stage of tauopathy.

### TOMA attenuated retinal pathologies in P301L mice

**Fig. 6:**
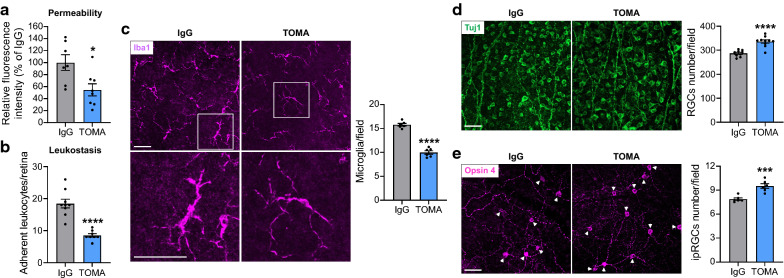
TOMA treatment alleviates retinopathies. TOMA or control IgG was intravenously injected to P301L mice at 1 month of age and 1 week before sample collection at 3 months of age. **a** Retinal permeability. n = 7–8/group. **b** Leukostasis. Bar graph represents the number of stationary leukocytes adherent to the retinal vasculature per retina. n = 8–9/group. **c** Microglia were stained with anti-Iba1 antibody (purple) and images were taken at the NFL-GCL by confocal microscopy. Squares in the upper panel of images are zoomed in to show microglial activation. Bar graph represents the number of microglia at the NFL-GCL. n = 6/group. **d**, **e** Representative images of retinal flatmounts labeled with Tuj1 antibody (green) for RGCs and Opsin 4 antibody (purple) for ipRGCs at the peripheral retina. Arrowheads indicate the soma of ipRGCs. Bar graphs represent the numbers of RGCs (n = 9–10/group) and ipRGCs (n = 6/group) per field; eight images were taken at the peripheral retina for each sample and calculated as average value. Scale bar: 50 µm. * *p* < 0.05; ***p < 0.001; ****p < 0.0001 versus IgG

Many studies suggest that tau oligomers (soluble and intermediate tau aggregates) but not neurofibrillary tangles (NFTs) formed by the deposition of aggregated tau are the main toxic tau species in tauopathies [23, 27], and systemic administration of TOMA reversed neurodegenerative phenotypes in the brain of aged P301L tau transgenic mice [9, 10, 22]. Since tau oligomers are present in the retina and the brain in P301L mice [51], we examined the effect of TOMA treatment on retinal pathology in P301L mice. We intravenously injected TOMA to mice at 1 month of age and 1 week before sample collection at 3 months of age, and analyzed retinal vascular and neuronal changes. Our study showed that TOMA treatment significantly attenuated retinal vascular permeability (~ 7.4-fold increase in P301L-IgG vs WT, ~ 4.1-fold increase in P301L-TOMA vs WT) and leukostasis (Fig. 6a, b). Immunostaining with anti-Iba1 antibody revealed that the retina from TOMA-treated P301L mice exhibited a decreased number of microglia in the NFL-GCL in comparison to that from IgG-treated P301L mice, and microglia morphology changed from amoeboid to a more ramified shape after TOMA treatment (Fig. 6c; Additional file 1: Fig. S8). The numbers of RGCs and ipRGCs were also higher in the retina of TOMA-treated P301L mice than control IgG-treated mice (Fig. 6d, e; Additional file 1: Fig. S9) and almost back to normal levels of WT retinas. Taken together, these results suggested that TOMA can be used to effectively attenuate retinal pathology during tauopathy.

## Discussion

The NVU is a complex structure consisting of endothelial cells, mural cells (pericytes, smooth muscle cells), astrocytes, neurons and microglia [28, 50]. NVU injury and dysfunction are recognized as a key player in neurodegenerative diseases such as AD, frontotemporal dementia, amyotrophic lateral sclerosis and dementia with Lewy Bodies [28]. The retina, as the only visually accessible tissue in the central nervous system (CNS), is an excellent platform to study NVU alterations in diseases and has attracted much interest to evaluate its potential use as a biomarker for neurodegenerative diseases. While these studies have found significant RGC loss, dysfunction and optic nerve degeneration in patients or animal models of neurodegenerative diseases and a few studies suggest microglial and glial activation in the retina at the early stage of the diseases [8, 12, 18, 20, 25, 35, 47, 64], retinal vascular changes are less known and there is a gap in conducting an integrated analysis addressing temporal alterations of different retinal cells in the NVU in these diseases. Since tau phosphorylation and aggregation is one of the major causes of neurodegeneration and correlates more closely with dementia status than Aβ deposition does [6], we systemically determined alterations of NVU in the retina using histological and non-invasive imaging and functional tests in two transgenic mouse models of tauopathy (P301S and P301L mice) at different stages of disease progression. Our studies unravel several NVU changes in the retina which were unappreciated previously.

RGCs are the only retinal neurons that directly connect to other neurons in the brain. They share more common features and pathological mechanisms with brain neurons than other retinal neurons and are affected during many neurodegenerative diseases in the CNS [35, 64]. P301S mice used in our study carry human tau gene with the P301S mutation, which is driven by the mouse prion promoter [65]. These mice develop microglial activation, synapse loss and impaired synaptic function in the hippocampal region at 3 months of age. However, there is no obvious reduction in the number of hippocampal neurons in 6-month-old P301S mice although severely impaired synaptic plasticity is observed at this stage [65]. In the retina of P301S, we found significant RGC loss and dysfunction have occurred at 3 months of age. This result is consistent with other AD-relevant mouse model in which RGC loss occurs earlier than neuronal loss in the brain [25]. One possibility is that the anatomical and metabolic features of RGCs may condition these cells more sensitive to aggregated proteins than neurons in the brain. Another possibility is that since the retina is a transparent tissue and RGCs are localized in the inner retina, it is much easier to accurately quantify the number of RGCs than that of brain neurons, which makes it possible to identify cell loss even when it is not dramatic. Interestingly, compared with total RGCs, the loss of ipRGCs was more prominent and occurred earlier. Since ipRGCs sense the light and play a major role in synchronizing circadian rhythms, it is possible that early loss of ipRGCs may at least partially attribute to circadian dysfunction seen in AD patients [36, 48]. Moreover, 20–30 subtypes of RGCs have been proposed based on their differences in physiological, morphological and molecular properties [46, 55]. A recent single cell transcriptome profiling further classifies RGCs into 40 subtypes using clustering algorithms [53]. Our data suggest that certain RGC subtypes may be preferentially affected by stresses induced by tauopathy, which is similar to the selective vulnerability in the nervous system that has been noticed in the brain during AD [19]. Future studies using immunolabeling with multiple markers, RNA in situ hybridization, and single-cell genomic and molecular profiling approaches are necessary to characterize differential actions of RGC subtypes in tauopathy. Considering the relatively simple neuronal network in the retina vs. brain, it makes the retina an appealing platform to understand the fundamental mechanisms underlying the selective vulnerability in the nervous system during neurodegenerative diseases. Of note, while previous studies using a Thy-1 promotor driven-P301S mouse strain showed a reduction of anterograde axonal transport in the optic nerve at 3 months of age and RGC dysfunction at 5 months of age [8, 20, 47], RGC loss was not obvious in this mouse strain [20]. One possibility is that the mouse prion promotor is stronger than the Thy-1 promotor and therefore P301S mice used in our study have more severe phenotype. Another possibility is that we used RGC-specific antibodies to specifically analyze RGC changes while Gasparini et al. used an antibody against NeuN [20] which is expressed on other neurons beside RGCs.

The non-neuronal cells in the NVU are critical for the survival and homeostasis of neurons and maintenance of their functions. In the CNS, neurons and vessels are functionally integrated, and neural activity and vascular dynamics are tightly coupled to meet the high demands of oxygen and glucose of neurons [3]. The tightly controlled blood–brain barrier (BBB) or blood–retina barrier (BRB) selectively moves molecules, ions, and cells between the blood and the CNS to ensure a homeostatic environment for proper neuronal function and protect them from toxins, pathogens and inflammation [3, 13]. BBB breakdown often occurs in neurodegenerative diseases including AD and contributes to neuronal dysfunction and degeneration [13]. BRB breakdown is a major cause of vision loss in diabetic retinopathy. Nevertheless, alterations of BRB integrity under AD-related conditions including tauopathy have not been investigated. Using FITC-BSA which is a more sensitive tracer than Evans blue, we provided the first evidence that increased vascular leakage occurred in the retinas of P301S and P301L mice. Analysis of VE-Cadherin and occludin in retinal vessels further revealed adherens and tight junctions were impaired in P301S mice. These studies demonstrated that loss of BRB integrity occurred at the very early stage of tauopathy (1 month of age), and such change appeared earlier than RGC loss and was associated with increased vascular inflammation as demonstrated by increase in leukocyte attachment to the vessels. Nonetheless, our OCT analysis did not find subretinal fluid accumulation which usually occurs when protein and fluid leaking from retinal or choroidal vessels overweighs fluid removal [14, 44], suggesting that the fluid drainage function of the retina is still intact in these animals. Overall, our study together with a recent report that pericyte loss is detected in the retinas from AD patients [59] highlights the similarity between the retina and the brain in which vascular pathological change is an early event in tauopathy. As vessels play a critical role in tau clearance during AD [62, 63], the abnormal vascular network may impair clearance of retinal tau and further accelerate retinal neuronal injury.

We also studied microglial and glial changes in the retina. Microglial activation has been observed in 3xTg mice that express three AD-related mutated genes (*PS1*M146 V, *APP*Swe, and P301L tau) [25, 54]. Using retinal flatmount staining that allows observing microglial changes more clearly than using retinal sections, together with co-staining with vessels, we discovered several new features that have not been previously described. We found that tauopathy itself was sufficient to drive microglial morphological changes in the retina in the absence of amyloidosis. Moreover, microglial recruitment and activation occurred at the very early stage of tauopathy and was associated with early vascular pathological changes. While microglia are present in three retinal layers, only cells located in the NFL-GCL exhibited changes in cell number and morphology, suggesting cells located in this layer are more sensitive to tauopathy. An increase in the interaction between microglia and vessels during tauopathy may reflect recruitment of microglia during vascular inflammation. Meanwhile, microglia may contribute to vascular inflammation and BRB breakdown by crosstalking to inflammatory cells [52] and vascular cells. In contrast to microglia, gliosis occurred at a relative late stage, similar to what occurs in the brain. Overall, the similar changes in vessels, microglia and glia between the retina and the brain strongly support the notion that the retina can be used as a “window” for early diagnosis of neurodegeneration in the brain during tauopathy since the optical clearance of retina offers it as an excellent platform for non-invasive imaging. This possibility was supported by our OCT analysis showing increase in the thickness of GCC likely reflecting increased vascular leakage in P301S and P301L mice as well as by the feasibility to examine microglial actions with non-invasive SLO after labeling them with fluorescent probes [32]. Certainly, as one biomarker is usually not sufficient for disease diagnosis, it is necessary to combine other approaches (e.g. analyzing tau oligomers and Aβ peptides in the plasma for AD, measuring intraocular pressure to exclude glaucoma, and measuring drusens under the retina to exclude age-related macular degeneration) to enhance the sensitivity and specificity of prediction when using retinal imaging to diagnose and monitor brain pathology in tauopathy. Of note, while the retina developed alterations of non-neuronal cells in the NVU similar to those in the brain during tauopathy, increased gliosis, leukocyte adhesion and infiltration, microglial recruitment and activation may not necessarily relate to vascular changes but rather consequences of activation of other pathological pathways in particular the inputs from stressed neurons. Further studies of cell–cell crosstalk in the NVU will help to address the underlying mechanisms and better understand this process.

Interestingly, while retinal neuronal injury has been noticed in tauopathy and other AD-related conditions, few studies have been performed to address this issue. As aggregated tau protein in particular the soluble tau oligomers is toxic and tau immunotherapy has been shown to be beneficial in various animal models [2, 23, 27], we investigated the effects of TOMA on retinal NVU in P301L mice previously used to evaluated the therapeutic effects of TOMA on neurodegenerative phenotypes in the brain, which allowed us to follow the treatment procedure of previous studies and also compare retinal changes to those changes reported in the brain [9, 10, 22]. Since TOMA provides protection on working memory for at least 2 months [10], we started to treat animals with TOMA at 1 month of age when RGC loss was not significant so that we could evaluate if it could prevent progressive RGC loss from 1 month of age to 3 months of age. To ensure effective treatment before sample collection, a second dose of TOMA was given at 7 days before sample collection. Our studies showed that TOMA treatment significantly attenuated retinal pathological changes, suggesting that tau immunotherapy could be potentially used to prevent vision loss in patients with tauopathy and improve their life quality considering that visual impairment, such as a decline in motion blindness, depth perception, color perception and contrast sensitivity, is common in AD patients [45]. Moreover, the comparable beneficial effects of TOMA on the retina and the brain [9, 10, 22] suggest that similar mechanisms of neurodegeneration are operating in both organs. Therefore, the retina may serve as an alternative platform to study mechanisms of neurodegeneration of the brain and to evaluate efficacies of agents developed to treat tauopathy.

## Conclusions

In summary, using mouse models of tauopathy, we demonstrate that the retina develops a series of pathological changes in the NVU similar to those in the brain, including RGC loss, vascular inflammation and barrier breakdown, and microglial and glial activation. Moreover, many of these changes appear earlier in the retina than those reported in the brain [65] and similar pathological mechanisms may be operating in both organs. A limitation of this study is that we did not compare pathological changes of the NVU in the retina and the brain side-by-side in the same mouse cohorts. Nonetheless, our results supports further investigation of using the retina as a potential site for the detection and quantification of several key biomarkers for tauopathy in the brain, study of molecular mechanisms of neurodegeneration, and examination of the crosstalk between neurons and vessels during tauopathy.

## Supplementary Information

**Additional file 1:** Supplementary Figures.

## Data Availability

All data generated or analyzed during this study are included in this published article and its supplementary information files.

## Abbreviations

AD: Alzheimer's disease
BBB: Blood–brain barrier
BRB: Blood–retina barrier
BSA: Bovine serum albumin
CNS: Central nervous system
Con A: Concanavalin A
ERG: Electroretinography
FITC: Fluorescein isothiocyanate
FTDP-17: Frontotemporal dementia with parkinsonism-17
GCC: Ganglion cell complex
GCL: Ganglion cell layer
GFAP: Glial fibrillary acidic protein
GFP: Green fluorescent protein
INL: Inner nuclear layer
IPL: Inner plexiform layer
ipRGC: Intrinsically photosensitive RGCs
IS: Inner segment
NFL: Nerve fiber layer
NFTs: Neurofibrillary tangles
NND: Nearest neighbor distance
NVU: Neurovascular unit
PBS: Phosphate-buffered saline
pSTR: Positive scotopic threshold response
RGCs: Retinal ganglion cells
SD-OCT: Spectral domain optical coherence tomography
SLO: Confocal scanning laser ophthalmoscope
TOMA: Tau oligomer monoclonal antibody
WT: Wild type
