# Supplementary material for: Early alterations of neurovascular unit in the retina in mouse models of tauopathy
Source: Acta Neuropathol Commun. 2021 Mar 24;9:51. doi: 10.1186/s40478-021-01149-y (PMC7992935; doi:10.1186/s40478-021-01149-y)
Supplement: Supplementary file 1 — Additional file 1: Supplementary Figures. [file 40478_2021_1149_MOESM1_ESM.pdf]

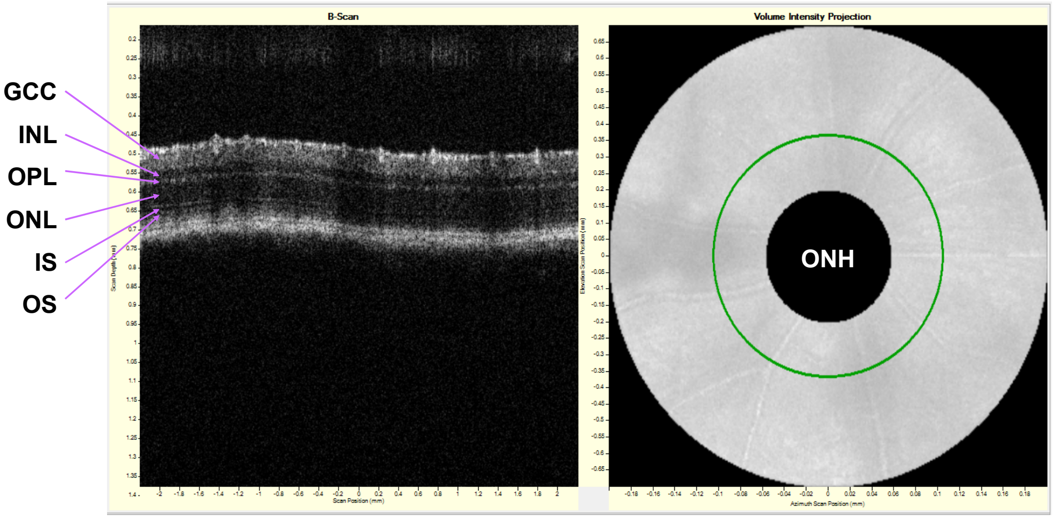

Supplementary Figure 1. Illustration of retinal thickness measurement by OCT. Right: The donut-shaped area of annular scan with the inner and outer radiuses which were 200 and 700 μm from the center of the optic nerve head (ONH), respectively. The green circle represents the position of one of the annular B-scans. Each B-scan consists of 1000 A-scans. 100 of such annular B-scans were performed to cover the whole donut-shaped area. Left: A representative annular B-scan that showed different retinal layers. GCC (ganglion cell complex) includes all three innermost layers: nerve fiber layer, ganglion cell layer and inner plexiform layer; INL: inner nuclear layer; OPL: outer plexiform layer; ONL: outer nuclear layer, IS: inner segment; OS: outer segment.

Supplementary Figure 1

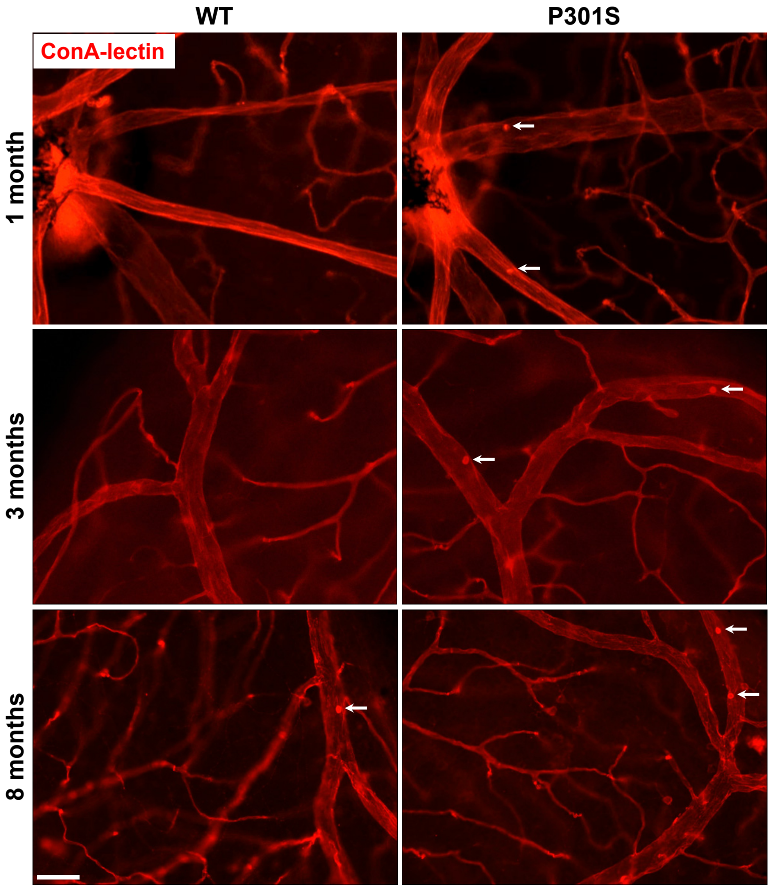

Supplementary Figure 2. Leukostasis is increased in the retina of P301S mice. WT and P301S mice were subjected to leukostasis assay at various ages. Representative images of leukostasis were shown. Red, Con A-labeled retinal vasculature and adherent leukocytes (indicated by arrows).

Supplementary Figure 2

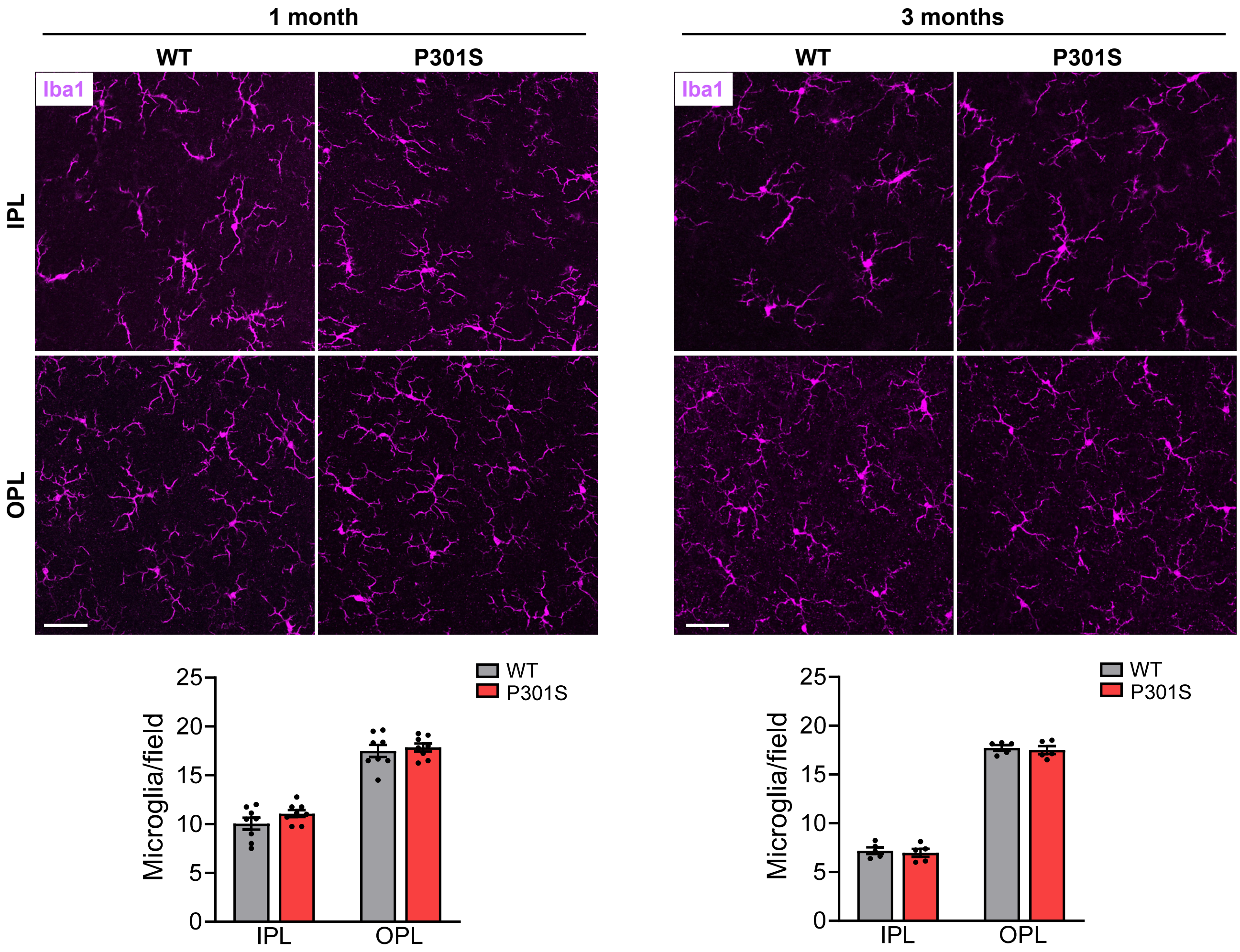

Supplementary Figure 3. Microglia recruitment/activation is not altered in the retinal IPL and OPL of P301S mice. Microglia were stained with anti-Iba1 antibody (purple) at 1 and 3 months of age. Images were taken at the IPL and OPL by confocal microscopy. Bar graphs represent the number of microglia at the IPL and OPL. Scale bar: 50 μm. n=5-8/group.

Supplementary Figure 3

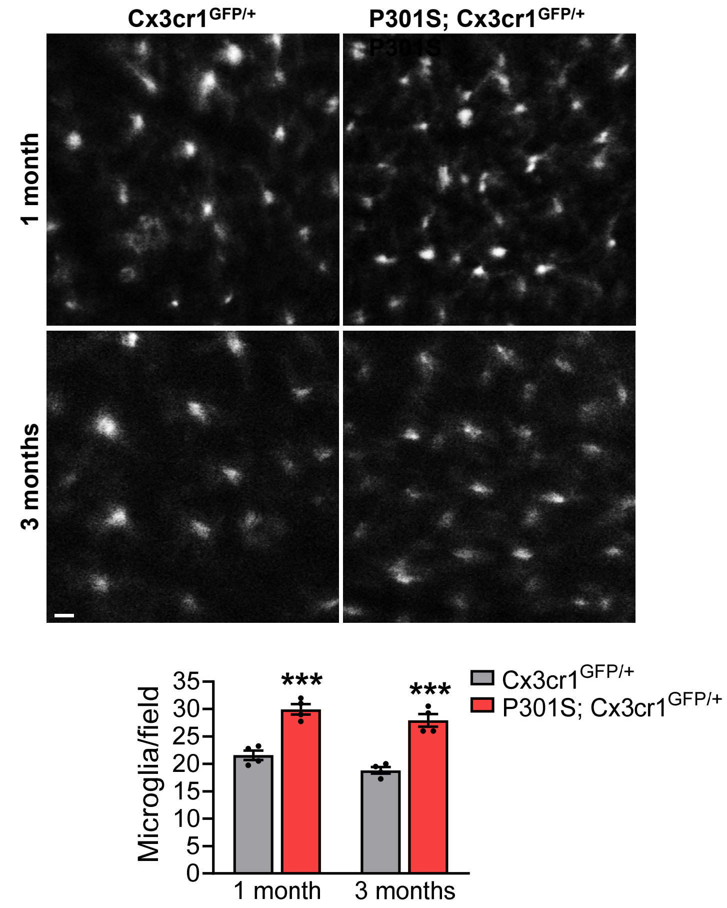

Supplementary Figure 4. Microglia is increased in the retina of P301S mice. Representative SLO images of the retinas of Cx3cr1$^{GFP/+}$ and P301S; Cx3cr1$^{GFP/+}$ at 1 and 3 months of age were shown and the number of microglia was counted. Scale bar: 200 μm. n=4/group. ***p<0.001 versus Cx3cr1$^{GFP/+}$.

Supplementary Figure 4

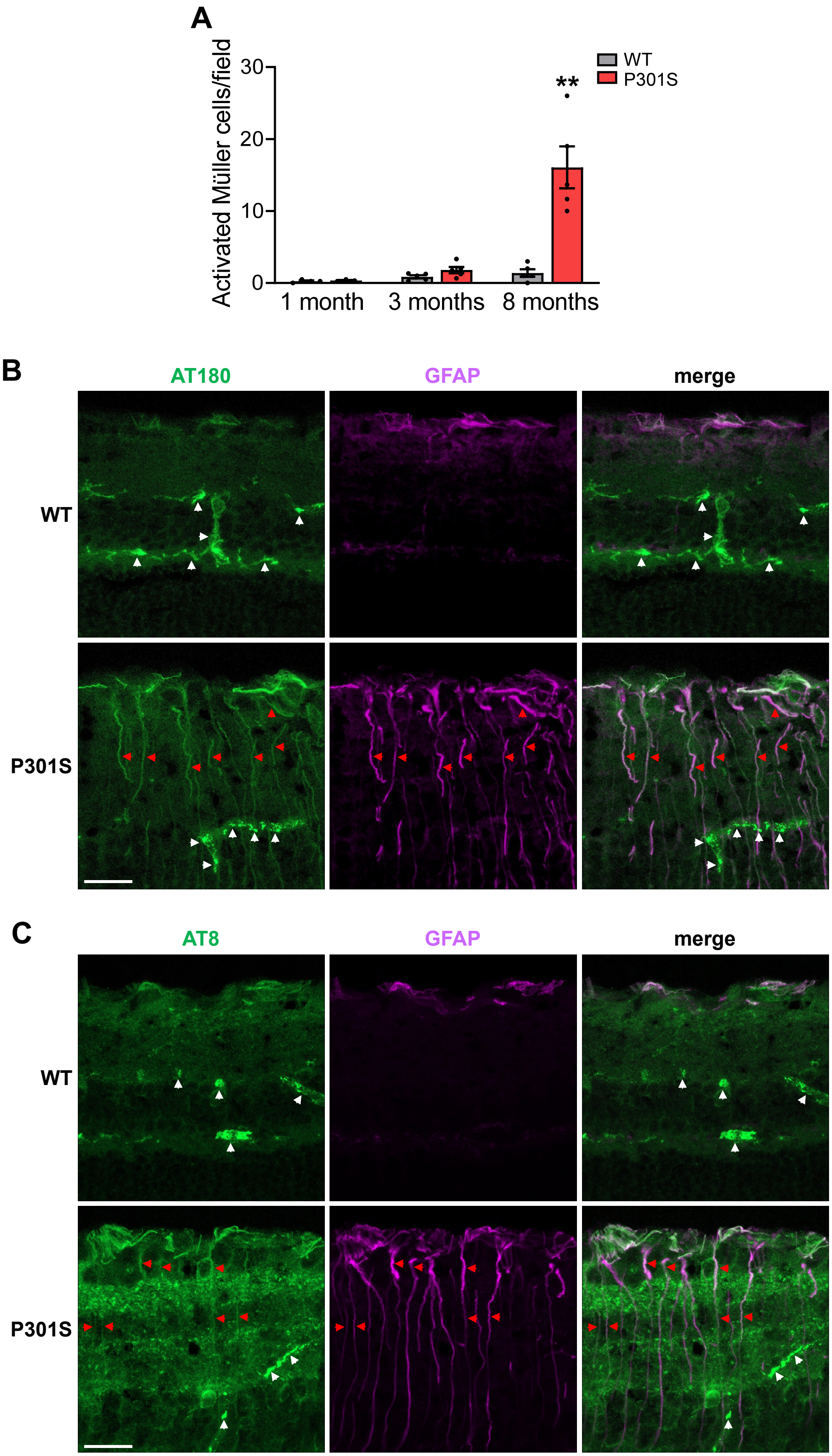

Supplementary Figure 5 . Characterization of Müller cells in P301S mice. (A) Graph represents the quantification of activated Müller cells in Figure 3E. n=3-5/group; **p<0.01 versus WT. (B) Phosphorylated tau (green) was stained with AT180 and AT8 antibodies and activated Müller cells was stained with antibody against GFAP (purple) in retinal sections from 8-month-old WT and P301S mice. Red arrowheads indicate phosphorylated tau in activated Müller cells. White arrowheads indicate non-specific staining. Scale bar: 20 μm. n=4/group.

Supplementary Figure 5

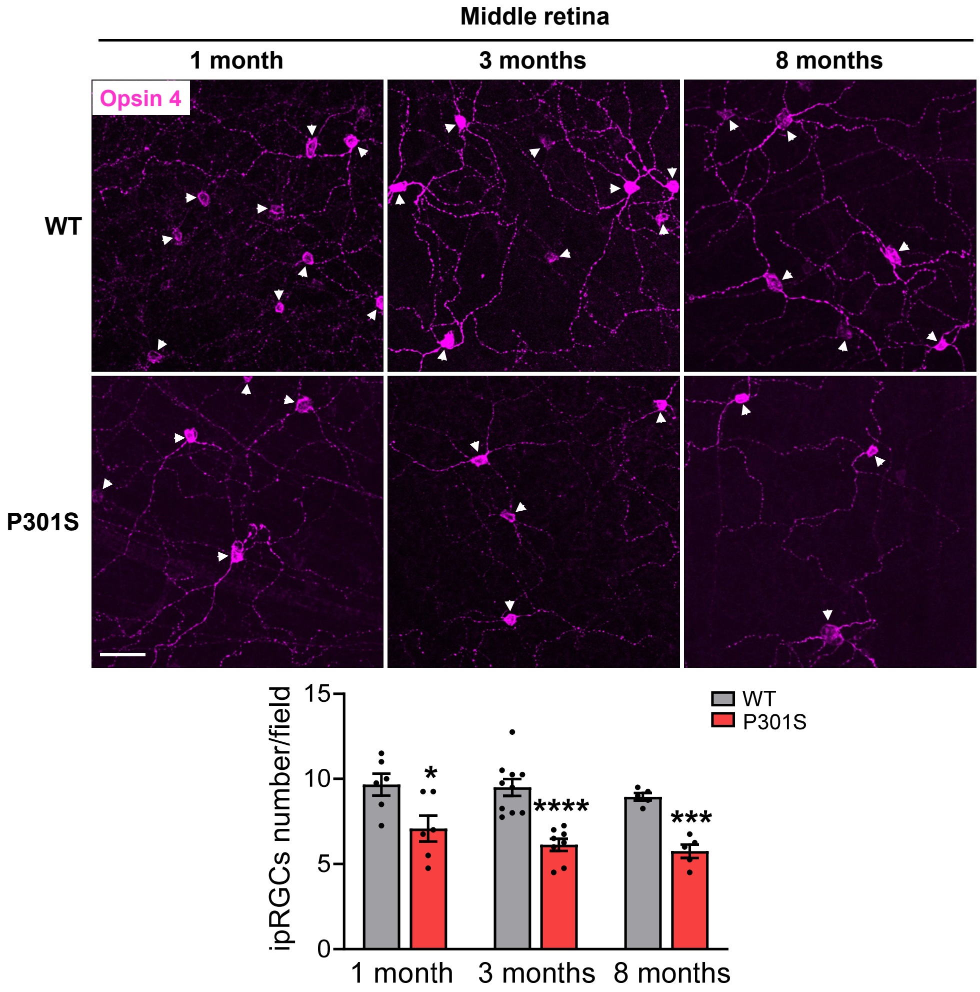

Supplementary Figure 6. ipRGCs are impaired in the middle retina of P301S mice. ipRGCs were stained with Opsin 4 antibody (purple) and the number of ipRGCs in the middle retina was quantified. Arrowheads indicate the soma of ipRGCs. Scale bar: 50 μm. n=5-10/group; eight images were taken in the middle retina and calculated as average value for each sample. *p<0.05, ***p<0.001, ****p<0.0001 versus WT.

Supplementary Figure 6

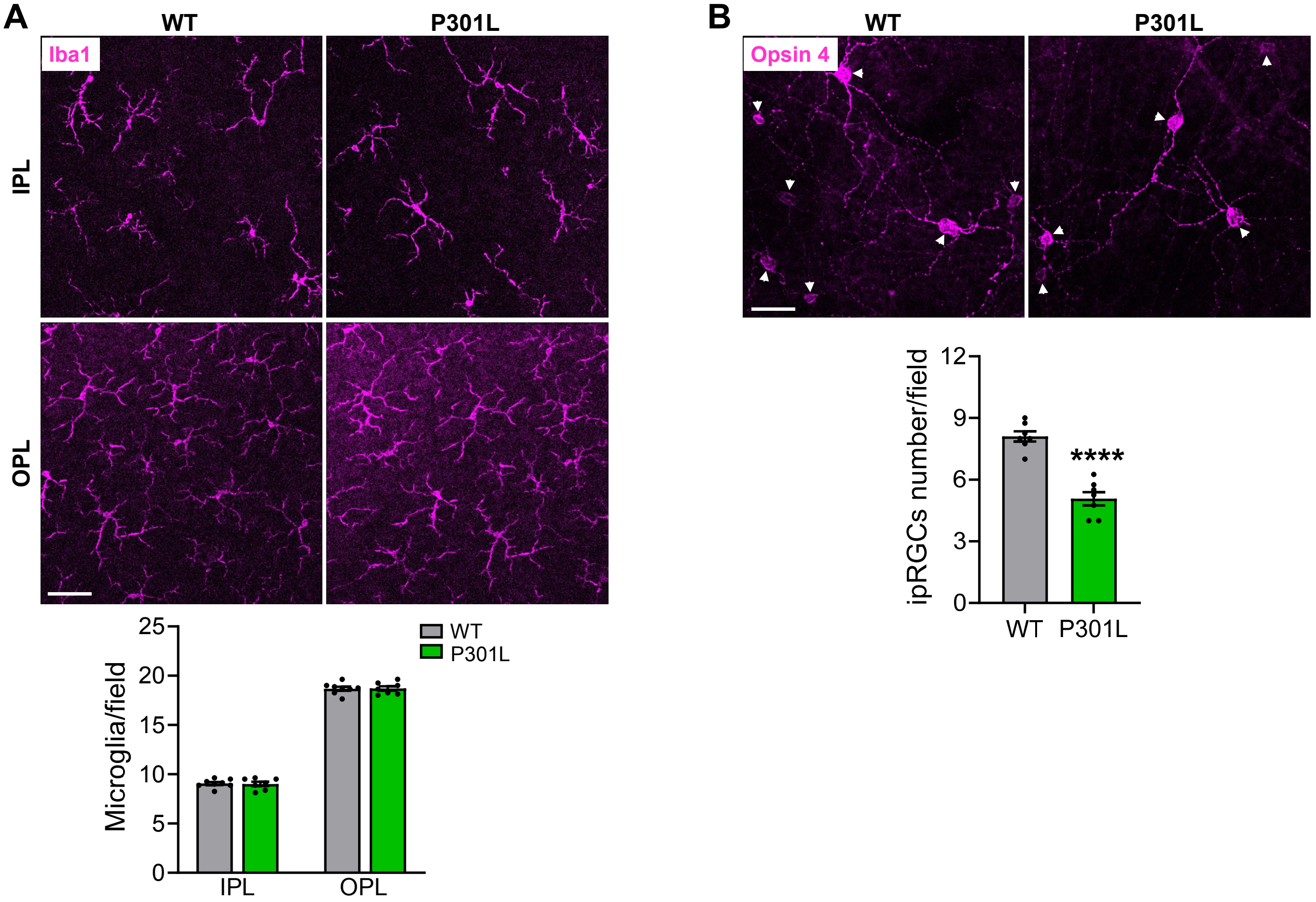

Supplementary Figure 7. Characterization of retinal pathogenesis in P301L mice. (A) Microglia recruitment/activation is not altered in the retinal IPL and OPL of P301L mice. Microglia were stained with anti-Iba1 antibody (purple) at 3 months of age. Images were taken at the IPL and OPL by confocal microscopy. Bar graph represents the number of microglia at the IPL and OPL. (B) ipRGCs are impaired in the middle retina of P301L mice at 3 months of age. ipRGCs were stained with Opsin 4 antibody (purple) and the number of ipRGCs in the middle retina was quantified. Arrowheads indicate the soma of ipRGCs. Scale bar: 50 μm. n=7-8/group; eight images were taken in the middle retina and calculated as average value for each sample. ****p<0.0001 versus WT.

Supplementary Figure 7

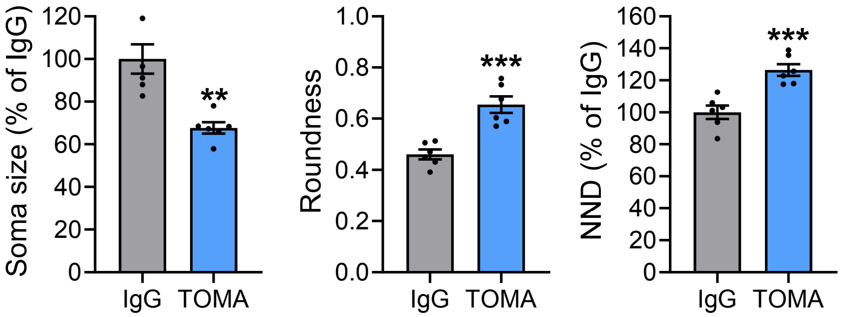

Supplementary Figure 8. Characterization of microglia in Figure 6C. Graphs represent the quantification of soma size and roundness, and nearest neighbor distance (NND) of microglia in the NFL-GCL of IgG or TOMA-treated P301L mice. n=6/group; **p<0.01 and ***p<0.001 versus IgG.

Supplementary Figure 8

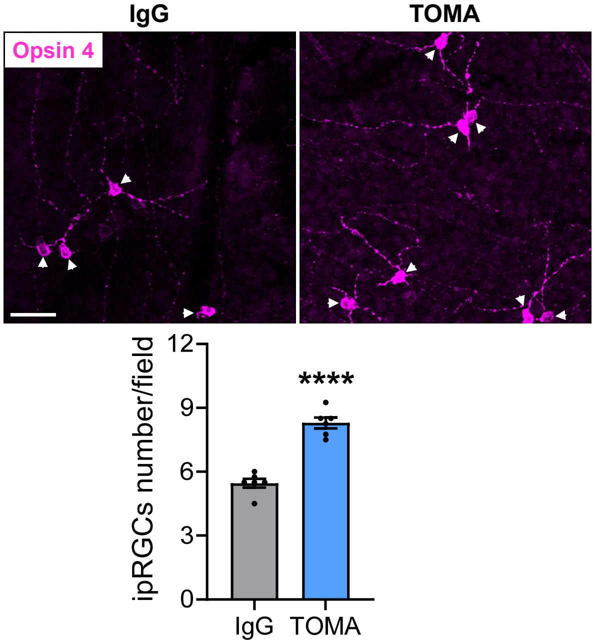

Supplementary Figure 9. TOMA treatment alleviates ipRGCs loss in the middle retina of P301L mice. ipRGCs were stained with Opsin 4 antibody (purple) and the number of ipRGCs in the middle retina was quantified. Arrowheads indicate the soma of ipRGCs. Scale bar: 50 μm. n=6/group; eight images were taken in the middle retina and calculated as average value for each sample. ****$p<0.0001$ versus IgG.

Supplementary Figure 9
